# Comprehensive analysis of GDFs as therapeutic targets and prognosis biomarkers in gastric cancer

**DOI:** 10.1097/MD.0000000000041976

**Published:** 2025-03-28

**Authors:** Minjie Zhu, Jiawei Hong, Xianfang Liu, Haiming Wang, Longquan Lou

**Affiliations:** a Department of General Surgery, Hangzhou Third People’s Hospital, Hangzhou, Zhejiang, China; b Department of Hepatobiliary and Pancreatic Surgery, Key Laboratory of Artificial Organs and Computational Medicine of Zhejiang Province, Shulan (Hangzhou) Hospital, Shulan International Medical College, Zhejiang Shuren University, Hangzhou, P.R. China; c Department of Cardiology, Zhejiang Provincial People’s Hospital, People’s Hospital of Hangzhou Medical College, Hangzhou, Zhejiang, China.

**Keywords:** bioinformatics analysis, biomarker, gastric cancer, GDF, immunotherapy

## Abstract

Growth/differentiation factors (GDFs, GDF1-3, GDF5-7, GDF9-11, and GDF15) belong to a subfamily of the transforming growth factor-β. GDFs play an important role in morphogenetic and developmental activities in many tissues. And many GDFs family numbers have been observed to be correlated with various types of tumors. However, the diverse expression patterns and prognostic values of ten GDFs in gastric cancer (GC) have yet to be analyzed. Herein we investigated the transcriptional and survival data of GDFs in patients with GC from the Gene Expression Profiling Interactive Analysis, The Cancer Genome Atlas, cBioPortal, Tumor Immune Estimation Resource, Tumor Immune Syngeneic Mouse, UALCAN, Human Protein Atlas Gene Expression Omnibus and The Database for Annotation, Visualization and Integrated Discovery databases. We found that multiple GDF family members are highly expressed in GC, which can prompt diagnosis and evaluate prognosis, and can be used as target points for GC immunotherapy.

## 1. Introduction

Growth/differentiation factors (GDFs) belong to a subfamily of the transforming growth factor-β (TGF-β) that contains 7 conserved cysteine residues forming a cysteine knot.^[1]^ As an inactive precursor protein, GDF 1 to 15 can be cleaved and assembled into active secreted homodimers.^[2]^ They play an essential role in a wide spectrum of morphogenetic and developmental activities in many tissues, such as the development of bones, kidneys and germ cells. The alterations of GDF family genes, especially GDF15 (macrophage inhibitory cytokine-1), have been observed to be correlated with various types of tumors including breast cancer, colorectal cancer, gastric cancer (GC),^[3,4]^ glioma^[5]^, and prostate cancer.^[6]^

GC, also called as stomach adenocarcinoma, is the fifth most prevalent cancer and the fourth leading cause of cancer-related death, where the rate of median survival is <12 months for the advanced stage.^[7–9]^ It is the result of a complex interplay of genetic and environmental factors, including *Helicobacter pylori* infection, age, high salt intake, low fruit and vegetable intake and increased use of antibiotics and acid suppressants.^[9,10]^

Due to the lack of specific signs of early GC and the low detection rate, most patients (>70%) have developed advanced-stage disease, and even had metastatic disease when first diagnosed with GC. These patients have lost the opportunity to undergo surgical resection. But due to the heterogeneity of GC, biomarkers used to predict prognosis have some limitations. Therefore, new biomarkers are of great significance as prognostic indicators in this field to effectively improve the prognosis and individualized treatment.

GDFs are a series of secreted ligand of the TGF-β. GDF1 and GDF11 involved in the establishment of left-right asymmetry in early embryogenesis and neural development.^[11,12]^ GDF2 regulates cartilage and bone development, angiogenesis and differentiation of cholinergic central nervous system neurons.^[13]^ GDF3 plays a role ocular and skeletal development.^[14]^ GDF5 participates in the development of numerous tissue and cell types, including cartilage, joints, brown fat, teeth, and the growth of neuronal axons and dendrites.^[15]^ GDF6 is required for normal formation of some bones and joints in the skull and axial skeleton.^[16]^ GDF7 helps stabilize neurons in the spinal cord.^[17]^ GDF9 participates in the regulation of ovarian reproductive hormone function.^[18]^ GDF10 can help neural repair after injury.^[19]^ GDF15 is involved in the stress response program of cells after cellular injury.^[20,21]^ GDF15 has the potential as a tumor marker and immunotherapy target for tumors such as colon cancer and lung cancer.^[22]^ GDF5, 7, 9, and 11 are also expected to be new immune targets for multiple tumor therapies.^[23–26]^ However, there are few studies on the expression and application prospect of GDFs family in GC.

**Figure 1.**
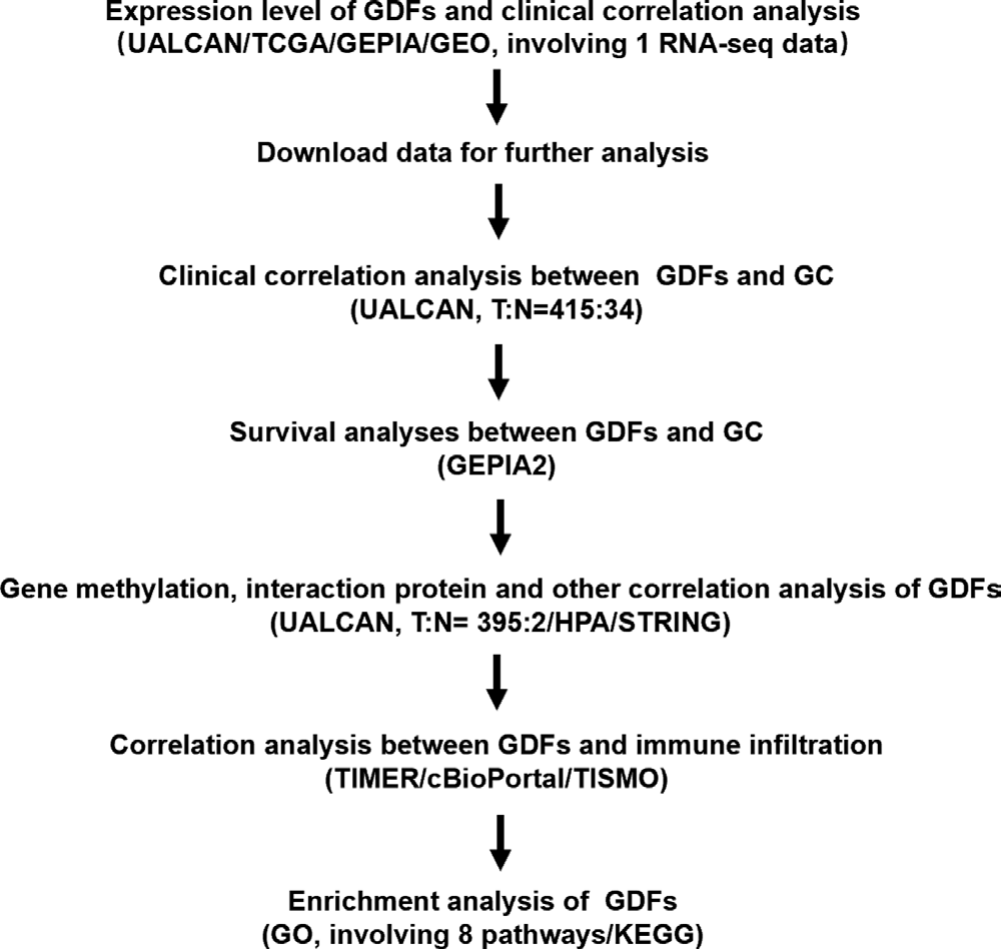
Study flow chart.

On the basis of the analyses of thousands of gene expressions or variations in copy numbers published online, we analyzed the expressions and mutations of different GDFs in GC patients in detail to determine the expression patterns, potential functions, and distinct prognostic values of GDFs in GC. A number of available databases, including Gene Expression Profiling Interactive Analysis (GEPIA), The Cancer Genome Atlas (TCGA), cBioPortal, Tumor Immune Estimation Resource (TIMER), Tumor Immune Syngeneic Mouse (TISMO), UALCAN, Human Protein Atlas (HPA) Gene Expression Omnibus (GEO), and The Database for Annotation, Visualization and Integrated Discovery were integrated to investigate the comprehensive effects of GDFs on GC. Figure 1 shows this study’s workflow.

## 2. Materials and methods

### 2.1. Data sources

All data analyzed in this study came from public databases, including TCGA, HPA, GEO, Genotype-Tissue Expression database, Clinical Proteomic Tumor Analysis Consortium, and Genomic Data Commons (GDC) data portal. All methods were performed in accordance with the reported criteria.

### 2.2. Gene expression analysis

UALCAN^[27,28]^ portal (http://ualcan.path.uab.edu/analysis-prot.html) is used to analyze the transcriptional levels and DNA methylation of the GDFs in GC and corresponding normal tissues option using data from TCGA (https://www.cancer.gov/tcga). The “Multiple Gene Comparison” module of GEPIA (Gene Expression Profiling Interactive analysis) webserver (http://gepia.cancer-pku.cn/) is used to analyze the comprehensive expression of GDFs in GC (using the “STAD” dataset) and normal tissues (Match TCGA normal and Genotype-Tissue Expression data). The “Pathological Stage Plot” module of GEPIA is used to analyze the relationship between the expression of GDFs and GC clinicopathological features. HPA webserver (http://www.proteinatlas.org/) is used to collect immunohistochemistry staining images of GDFs protein expression between GC and normal tissues. The RNA-seq data (GSE51575) is obtained from the GEO database to validate the clinical correlation of GDFs in GC. RNA-sequencing expression (level 3) profiles of GDFs genes in GC were downloaded from the TCGA dataset. The multi-gene correlation heatmap^[29]^ is displayed by the R software (version 4.0.1, https://www.R-project.org/). Spearman correlation analysis to describe the correlation between quantitative variables without a normal distribution.

### 2.3. Survival prognosis analysis

The “Survival Map” module of GEPIA2 is used to obtain the OS (overall survival) and DFS (disease-free survival) significance map data of GDFs in GC. Then, the “Survival Analysis” module of GEPIA2 is used to visualize each survival plots in the Survival Map.

### 2.4. Protein–protein interaction network

The online tool for the retrieval of interacting genes (STRING, https://string-db.org)^[30]^ was applied to establish a protein–protein interactions of GDFs and most frequently altered neighboring genes. GeneMANIA online database (https://genemania.org/) was used to analyze the co-expression networks and main functions of GDFs.^[31]^

### 2.5. Tumor immune analysis

TIMER web (http://timer.comp-genomics.org/) is a reliable, intuitive tool that provides systematic evaluations of the infiltration of different immune cells and their clinical impact.^[32,33]^ In our study, “Gene module” was used to evaluate the correlation between GDFs level and the infiltration of immune cells. “Survival module” was used to evaluate the correlation among clinical outcome and the infiltration of immune cells. “SCNA” module was used to analyze the comparison of tumor infiltration levels among GC with different somatic copy number alterations for GDFs. SCNAs including deep deletion, arm-level deletion, diploid/normal, arm-level gain, and high amplification.

cBioPortal (http://www.cbioportal.org/) was used to analyze the relationship between GDFs family and gene expression at different immune test sites in GC.^[34]^

“Gene” module of TISMO webserver (http://tismo.cistrome.org/) was used to compare GDFs expression levels in GC model (YTN16), between pre- and post-ICB treatment and responders and nonresponders.^[35]^

### 2.6. Enrichment analysis

Gene ontology (GO) analysis (biological processes, molecular function and cellular component) and Kyoto Encyclopedia of Genes and Genomes pathway enrichment were analyzed using R (version 4.0.1, https://www.R-project.org/) package clusterProfiler (version 3.16.0).^[36]^

### 2.7. RNA extraction and quantitative real-time PCR assay

Total RNA was extracted using the FastPure Cell/Tissue Total RNA Isolation Kit V2 (#RC112-01, Vazyme Biotech, Nanjing, China) in strict adherence to the protocols provided by the manufacturer. The RNA extracted from each sample was subjected to reverse transcription employing the HiScript II Q RT SuperMix for qPCR (#R222-01, Vazyme, China). Subsequently, RNA expression levels were quantified utilizing the ChamQ Universal SYBR qPCR Master Mix (#Q711-02, Vazyme, China) based on the manufacturer’s specifications on the Bio-Rad QX100 Droplet Digital PCR system (USA). Relative RNA quantification was determined using the 2-ΔΔCt method, with normalization to GAPDH. All primers were obtained from Tsingke Biotech Co., Ltd. (Beijing, China), and their sequences are listed here:

- GAPDH: forward: 5′-AAGGTGAAGGTCGGAGTCAAC-3′, reverse: 5′-GGGGTCATTGATGGCAACAATA-3′;
- GDF9: forward: 5′-ATGGCACGTCCCAACAAATTC-3′, reverse: 5′-ACTCAGCACTAGCAGCAATCT-3′;
- GDF15: forward: 5′-GACCCTCAGAGTTGCACTCC-3′, reverse: 5′-GCCTGGTTAGCAGGTCCTC-3′;

### 2.8. Western Blot and immunoprecipitation analysis

Total tumor proteins from various GC tissues were extracted RIPA buffer (Thermo Scientific, Waltham, MA, USA) after grinding, enriched with phosphatase inhibitors (1:100; Thermo Scientific, Waltham, MA, USA), and incubated for 60 minutes at 4 °C. Protein concentrations were determined using a BCA Protein Assay Kit (Pierce, USA). Subsequently, the samples were subjected to denaturation at elevated temperatures, cooled, and electrophoresed on 4% to 20% SurePAGE gels (15 wells) (GenScript, China), followed by transfer onto polyvinylidene fluoride membranes. The membranes were incubated with the specified primary antibodies, GDF9: #A2739, GDF15: #A22097, and TGF-β1: #A22296 from ABclonal Biotechnology Co., Ltd. (Wuhan, China) overnight, washed thrice with TBST (0.1% [v/v] Tween 20 in TBS), incubated with secondary antibodies, and developed using enhanced chemiluminescence. The detection of protein expression in animal tissues is similar to that described above.

### 2.9. TGF-β1 enzyme-linked immunosorbent assay

TGF-β1 was also analyzed by enzyme-linked immunosorbent assay (ELISA) using Human/Rat/Monkey/Porcine/Bovine Transforming Growth Factor Beta 1 ELISA Kit (#RK00055) (ABclonal) as described in the manufacturer’s directions. And all the study and clinical sample collection protocol was approved by the Research Ethics Board of the Hangzhou Third People’s Hospital.

## 3. Results

### 3.1. Transcription levels of GDFs in gastric cancer

**Figure 2.**
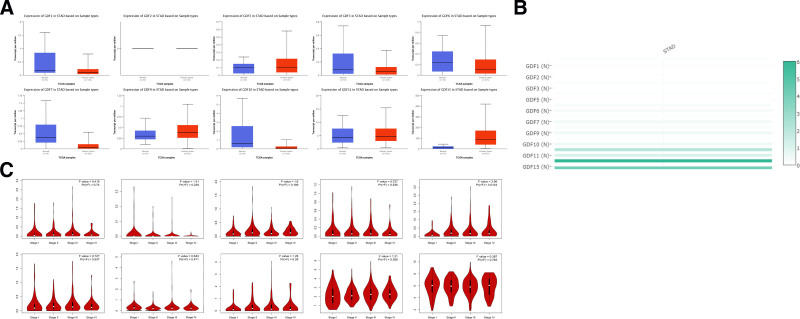
GDFs expression in gastric cancer. (A) Expressions of GDFs between gastric cancer and normal tissues; (B) Comprehensive expression of GDFs in gastric tissues; (C) Gastric cancer GDFs expression in different stages.

Ten GDF factors have been identified in mammalian cells, including in GDF1–3, GDF5–7, GDF9–11, and GDF15. The transcriptional levels of the GDFs were analyzed in GC and normal tissues using the UALCAN databases. As shown in Fig. 2A, GDF15 (*P* = 1.62E‐12), GDF11 (*P* = 1.38E‐2), GDF9 (*P* = 7.2811E‐3), and GDF3 (*P* = 4.95E‐6) were significantly up-regulated in primary tumor tissue (n = 415) compared with that in normal tissue (n = 34). In contrast, GDF7 (*P* = 2.911E‐2) and GDF10 (*P* = 2.4E‐3) were significantly down-regulated in primary tumors. Moreover, the other 3 members, GDF1, GDF5, and GDF6, did not show differential expression between GC tissues and normal tissues. In addition, GDF2 was not signally expressed in normal and tumor gastric tissues. Using the GEPIA dataset, the comprehensive expression levels of GDFs in gastric tissue is shown in Fig. 2B. Among all GDFs we evaluated, the relative expression of GDF15 was the highest.

We further explored the correlation among GDFs expression levels and clinicopathological parameters of GC patients using the “Pathological Stage Plot” module of GEPIA. GDF6 groups significantly varied (*P* = .0144), whereas GDF1–3, GDF7, GDF9–11, and GDF15 groups did not significantly differ (Fig. 2C).

**Figure 3.**
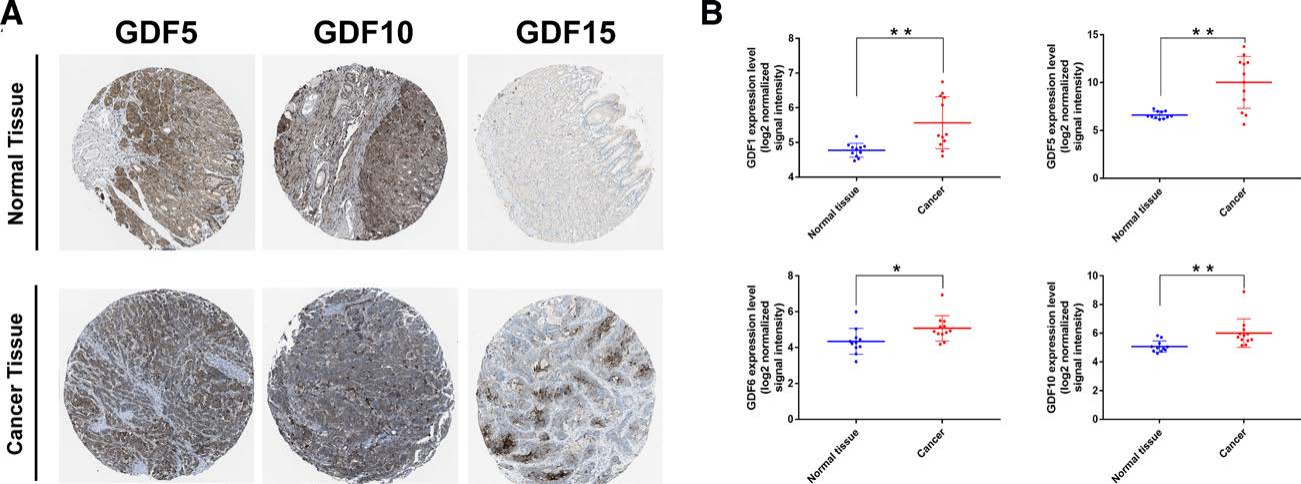
Clinical data validation of GDFs expression in gastric cancer. (A) Pathological detection of GDF5, 10, 15 expressions between gastric cancer and normal tissues; (B) GEO data validation of GDF5, 10, 15 expressions in gastric cancer and normal tissues.

We collected the data of HPA and GEO dataset (GSE51575) to verify the expression of GDFs in GC. As shown in Fig. 3, the case section specimens and clinical data do suggest that GDFs is highly expressed in GC.

### 3.2. Correlation between GDFs expression and the prognosis of patients with gastric cancer

**Figure 4.**
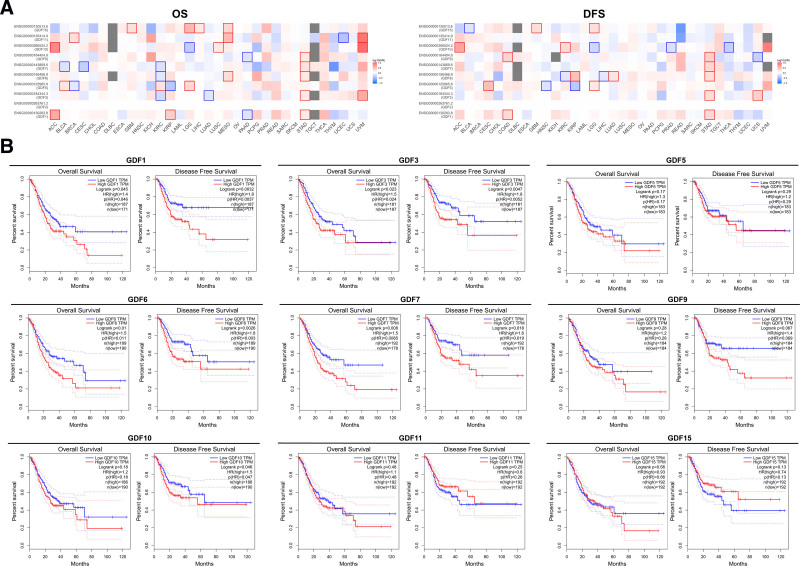
The relationship between the prognosis of GC patients and GDFs expressions. (A) GDFs’ OS and DFS in different cancers; (B) GDFs’ OS and DFS IN gastric cancer patients.

We used the datasets of TCGA and GEO to identify the possible prognostic value of GDFs expression in GC. We found highly expressed GDF1, 3, 6, 7, and 9 was linked to poor prognosis of OS and DFS (*P* < .01, Fig. 4A) for GC. Meanwhile, with regard to GDF5, 10, and 11, their expression levels had no significant effect on both the OS and DFS of GC patients.

Furthermore, analysis of the survival data using the Kaplan–Meier plotter tool presented the correlation between the mRNA levels of GDFs and the survival of patients with GC. As for GDF1, 3, 6, and 7, their median expression levels were observed to have a significant negative effect in both OS and analysis (*P* < .05, Fig. 4B). As for GDF9, its median expression levels had no effect on the OS (*P* = .28) and DFS (*P* = .067) of patients. Above all, in addition to GDF9, the other GDFs family members related to GC, including GDF1, 3, 6, and 7 can be considered as prognostic biomarkers for GC.

### 3.3. Predicted functions and pathways of GDFs changes and DNA methylation in gastric cancer

**Figure 5.**
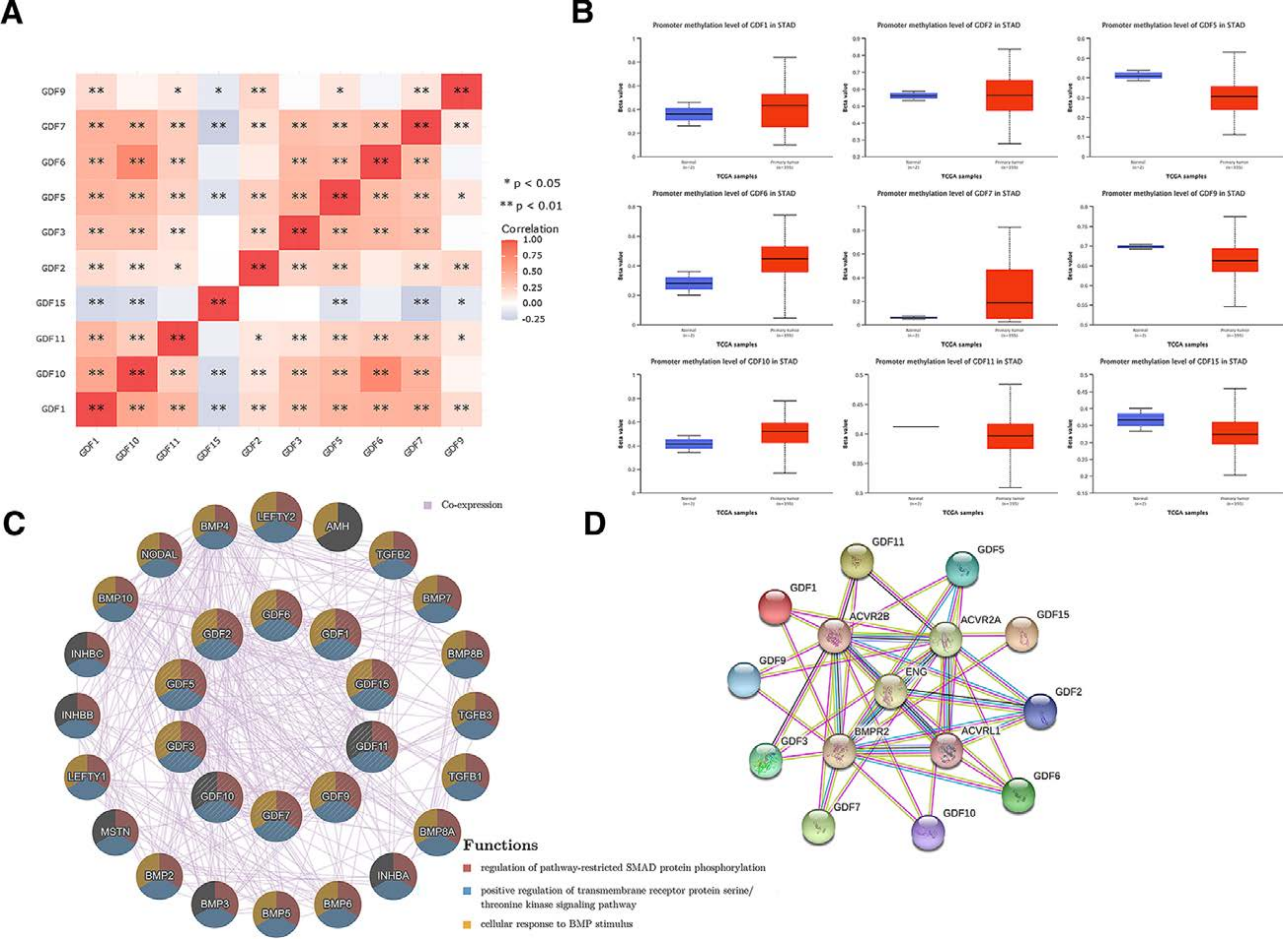
GDFs co-expression, methylation and interaction protein analysis. (A) Heatmap of GDFs co-expression with each other. ** *P* < .01, * *P* < .05; (B) GDFs’ methylation level in gastric cancer; (C) GDFs’ interaction protein analysis; (D) GDFs’ interaction protein enrichment analysis.

We calculated the relations of GDFs with each other by analyzing their mRNA expressions for gastric invasive carcinoma. The results indicated that there are significant positive correlations in the following GDFs: GDF1 with GDF5, GDF6, GDF7, GDF10, and GDF11; GDF3 with GDF5, GDF6, GDF7, and GDF10; GDF5 with GDF1, GDF3, GDF6, GDF7, and GDF10; GDF6 with GDF1, GDF3, GDF5, GDF7, and GDF10; GDF7 with GDF1, GDF3, GDF5, GDF6, and GDF10; GDF10 with GDF1, GDF3, GDF5, GDF6, and GDF7; GDF11 with GDF1 (Fig. 5A).

We chose the UALCAN approach to investigate the potential association between individual GDFs DNA methylation and the pathogenesis of GC in the TCGA project. Compared with normal tissues, we observed a reduced methylation level of GDF11 in tumor tissues for selected probes (Fig. 5B).

GDFs showed the complex network with the co-expression of 38.29%. Regulation of pathway-restricted SMAD protein phosphorylation, positive regulation of transmembrane receptor protein serine/threonine kinase signaling pathway, and cellular response to BMP stimulus, were identified as the main function of those genes (Fig. 5C). We further constructed the network of GDFs and the most frequently interacting neighboring genes, of which endoglin, bone morphogenetic protein receptor type 2 (BMPR2), activin A receptor like type 1 (ACVRL1), activin A receptor type family (ACVR), member 2A(ACVR2A) and 2B (ACVR2B) ranked among the top 5 genes (Fig. 5D).

### 3.4. GDFs expression is correlated with immune-infiltration level

**Figure 6.**
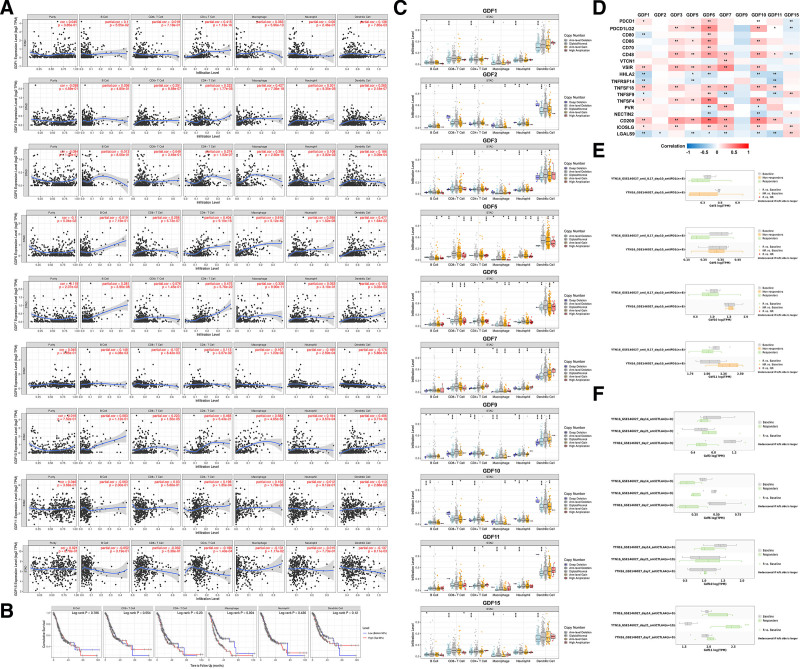
GDFs-related immune analysis. (A) The correlation between GDFs and immune infiltration; (B) immune-cell infiltration survival curve; (C) the relationship between GDFs copy number variation and the infiltration level of immune cell; (D) the co-expression relationship between GDFs and immune checkpoint; (E) effect of anti-PD1 treatment in gastric cancer mice with differential expression of GDFs; (F) effect of anti-CTLA4 treatment in gastric cancer mice with differential expression of GDFs.

The TIMER was employed to further investigate the immune infiltration of GC. The results in Fig. 6A showed that GDF1 and GDF2 were positively correlated with CD4+ T cells, B cells, and dendritic cells in GC. GDF3 was negatively correlated with cluster of B cell. GDF6 and GDF7 were positively correlated with B cells, CD4+ T cells, macrophage and dendritic cells and GDF10 was positively correlated with B cells and dendritic cells. while GDF15 was negatively correlated with cluster of CD8+/4+ T cells and dendritic cells. Next, we analyzed the relationship between different immune cell infiltration levels and the survival of GC patients. The high level of macrophage infiltration might lead to a worse prognosis (*P* = .004, Fig. 6B). Further, the SCNA exploration compared tumor infiltration levels among tumors with different somatic copy number alterations for GDFs (Fig. 6C). Also, we analyzed the relationship between GDFs family and gene expression at different immune test sites in GC with cBioPortal. As we can see, GDF3, 6, and 7 were positively correlated with the expression of multiple immune test sites which also confirmed the role of GDFs family in GC immunity microenvironment (Fig. 6D). Last but not least, the TISMO data demonstrated that the differential expression of GDF3, 6, and 11 in GC may contribute to the anti-CTLA4 (not anti-PD1) immunotherapy of GC which may require further validation in large patients’ immunotherapy data (Fig. 6E and F).

### 3.5. Bioinformatic analysis of GDFs

**Figure 7.**
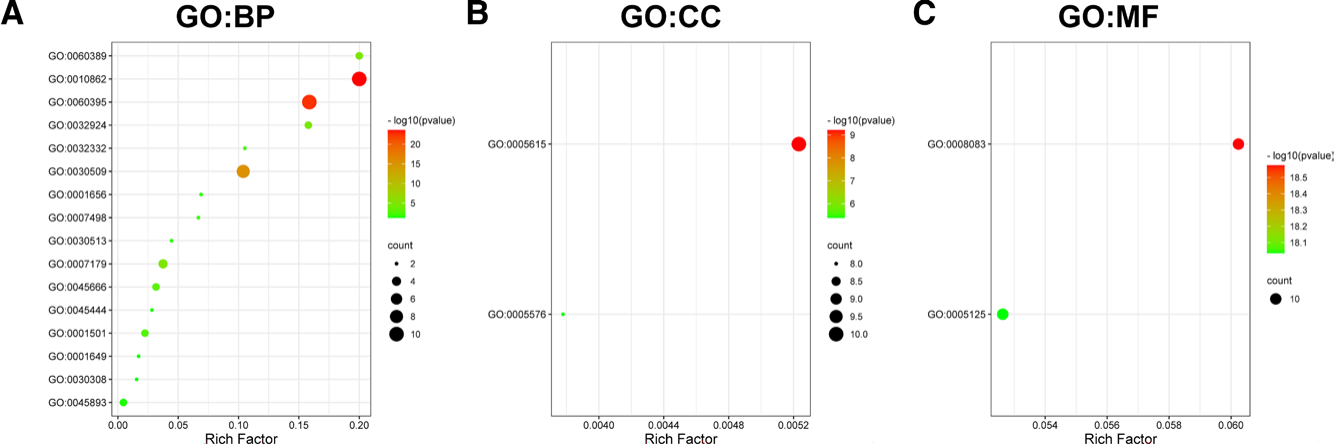
Enrichment analysis of GDFs. (A) GDFs’ enrichment analysis on biological processes; (B) GDFs’ enrichment analysis on cellular component; (C) GDFs’ enrichment analysis on molecular function.

**Figure 8.**
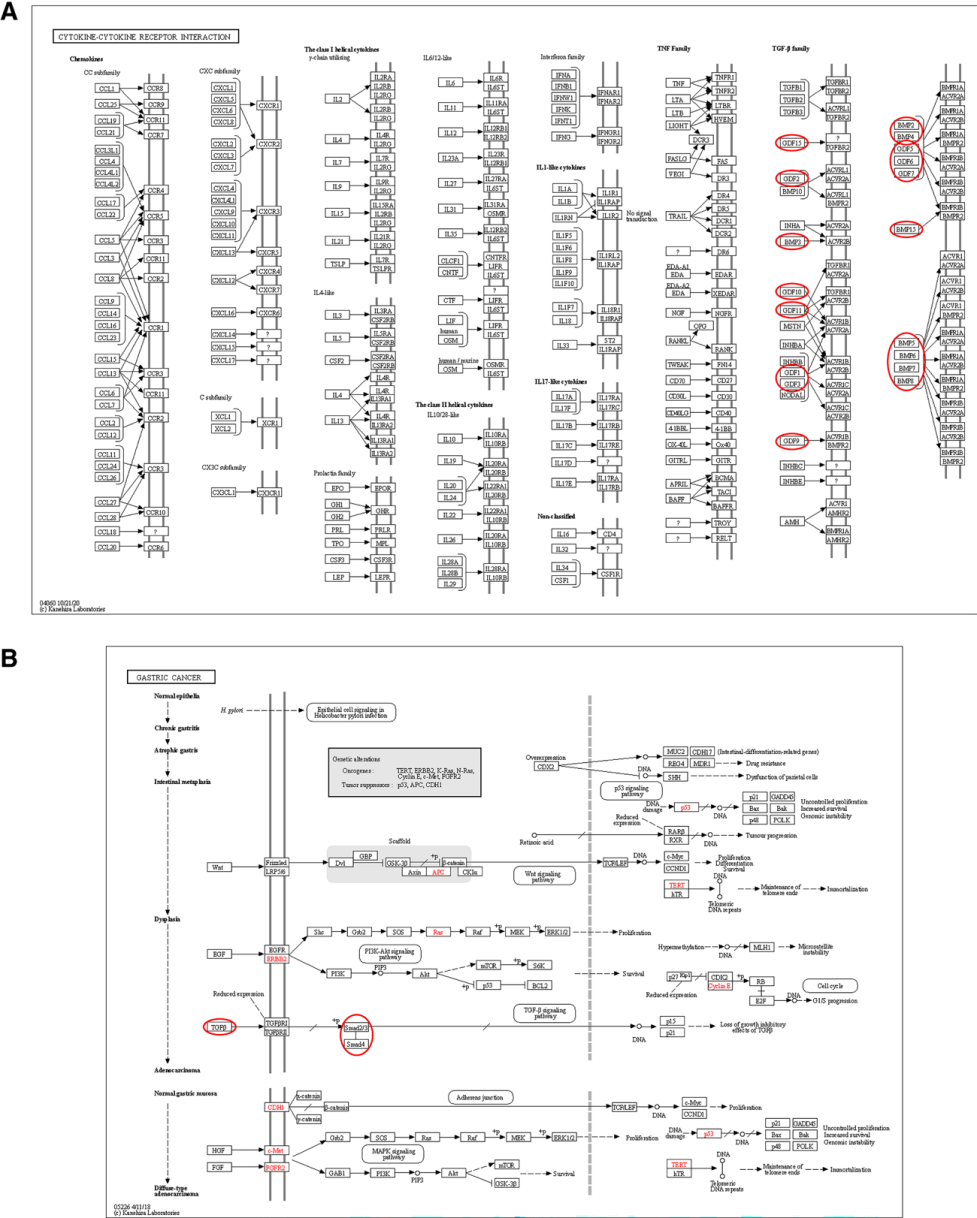
KEGG pathway analysis of GDFs. (A) Cytokine–cytokine receptor interaction pathway; (B) gastric cancer oncogenesis pathway. KEGG = Kyoto Encyclopedia of Genes and Genomes.

A further GO analysis showed that these GDFs were significantly enriched in SMAD transduction (GO:0060395) and phosphorylation(GO:0010862), BMP signaling pathway (GO:0030509), transforming growth factor beta receptor signaling pathway (GO:0007179), neuron differentiation (GO:0045666), and skeletal system development (GO:0001501) in the biological processes (BP) and enriched in growth factor activity (GO:0008083) and cytokine activity (GO:0005125) in molecular function (Fig. 7A–C). Among these pathways, the phosphorylation of SMAD signaling pathway was involved in the tumorigenesis and pathogenesis of GC (Fig. 8).

### 3.6. Clinical sample validation

**Figure 9.**
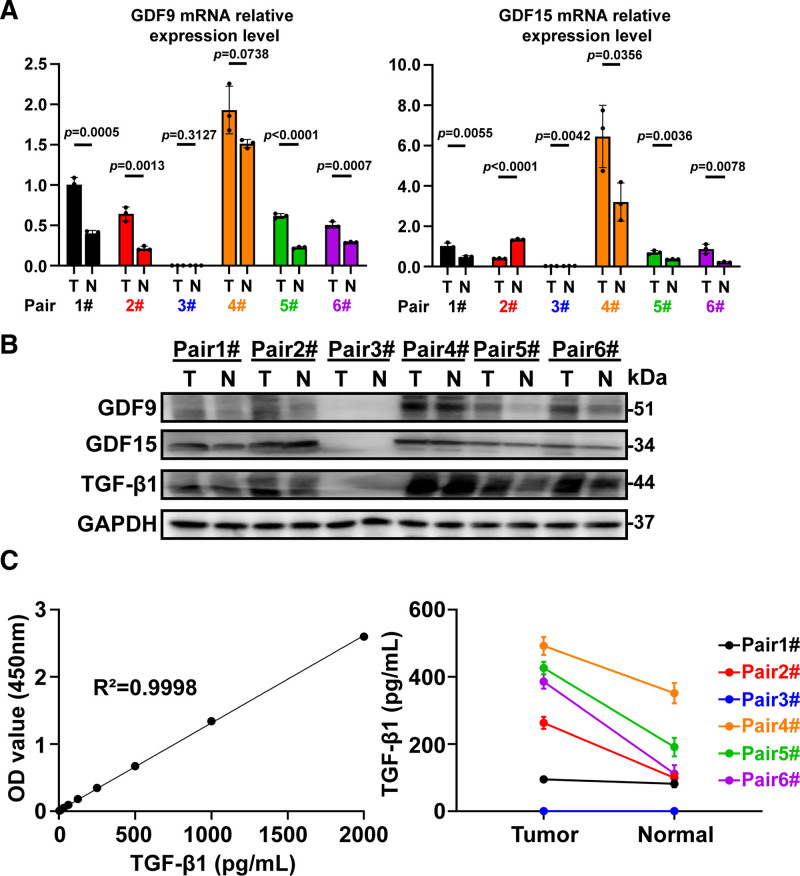
Six pairs of gastric cancer samples’ validation of GDFs. (A) mRNA expression of GDF9 and GDF15 in gastric cancer samples of 6 pairs was detected; (B) protein expression of GDF9, GDF15, and TGF-β1 in gastric cancer samples of 6 pairs was detected; (C) standard curve using the TGF-β1 standard sample in the ELISA kit. Subsequently, the expression level of TGF-β1 in 6 pairs of gastric cancer samples was calculated. ELISA = enzyme-linked immunosorbent assay.

Finally, we conducted further confirmatory analysis of GDF expression using GC and adjacent normal gastric tissue samples from our center. The results showed that, compared to normal gastric tissues, GC tissues exhibited higher mRNA and protein expression levels of GDF9 and GDF15 (Fig. 9A and B). Additional ELISA experiments confirmed that these clinical samples of GC had elevated expression levels of TGF-β1(Fig. 9C, R^2^ = 0.9998 in standard curve). This conclusion is consistent with the results of the above bioinformatics analysis.

## 4. Discussion

GDFs factor dysregulation has been reported in many cancers.^[22–26]^ Although the role of GDFs in the tumorigenesis and prognosis of several cancers has been partially confirmed, further bioinformatics analysis of GC has yet to be performed. The present study is the first study to explore the gene expression and prognostic values of different GDFs in GC. We hope that this research will contribute to improve treatment designs and enhance the prognosis accuracy of GC.

Among the GDFs, GDF15 is the most studied in various cancers, including hepatocellular carcinoma,^[37]^ melanoma,^[38]^ colorectal,^[39]^ ovarian,^[40]^ non-small cell lung cancer,^[41]^ gastric,^[42]^ and oral cancers.^[42]^ GDF15 is related to body weight metabolism through GFRAL (GDNF receptor alpha-like) receptor. GDF15 can trigger cancer-related cachexia by activating mitogen activated protein kinase 11 (MAP3K11).^[43]^ And GDF15 plays a role in many metabolic diseases such as type 2 diabetes and anorexia.^[21]^ Also, GDF15 participates in the regulation of immune homeostasis by immune activators and plays a role in inflammation, metabolic syndrome and autoimmune diseases.^[21]^ Our research confirmed that GDF15 is highly expressed in GC tissue and inhibits the infiltration of T cells and DC cells which has potential value as tumor prognostic marker.

Until now, little was known about the expression and role of GDF1 in cancer cells. Wei Cheng and colleagues confirmed that GDF1 can inhibit the excessive proliferation of hepatocellular carcinoma, but can promote its metastasis through activating the activin receptor-like kinase 7 (ALK7).^[44]^ One research found that the epigenetic silencing of GDF1 leads to the occurrence of GC.^[45]^ While our report verified that high GDF1 expression was significantly correlated with poor OS and DFS that it can be considered as prognostic biomarkers for GC. Kyoto Encyclopedia of Genes and Genomes analysis suggested that GDF1 can promote tumorigenesis by activating ACVR pathway. The role of GDF1 in GC deserves more in-depth study in the future.

The expression of GDF3 is different in various tumors. It is reported negatively regulated in breast cancer^[46]^ while positive regulated in retinoblastoma.^[47]^ We found GDF3 is highly expressed in GC cells. A higher GDF3 expression was correlated with poorer OS and DFS in GC patients. GDF3 is negatively correlated with the infiltration of B cells and positively correlated with the expression of multiple immune detection site genes. Those results made GDF3 as a potential GC biomarker and target for immunological therapy.

GDF5 overexpression is an oncogenic event in many types of cancers.^[26,48]^ GDF5 is a key regulatory factor in EB virus associated GC.^[48]^ Clinical data confirmed that GDF5 might have higher expression in GC tissues in GEO. A higher GDF5 expression was correlated with poorer OS in all of the patients with GC, but with no significance.

GDF6 is found abundant in several tumors. The BMP signal pathway induced by GDF6 was involved in Ewing Sarcoma, melanoma, prostate cancer, thyroid carcinoma and colorectal cancer.^[49–53]^ In this study, the expression of GDF6 increased with the improvement of GC stage. A higher GDF6 expression was correlated with poorer OS and DFS in all of the patients with GC. And its expression level was positively correlated with the infiltration of B cells, CD4+ T cells, macrophages and DC cells. There was no positive correlation with gene expression in multiple immune tests. Multiple positive correlations with immune regulation make GDF6, like GDF3, an appropriate biomarker and potential target for immunotherapy of GC.

GDF7, a member of the GDFs family of TGF-β, is abundant in multiple cancers including endometrial cancer,^[54]^ medulloblastoma,^[55]^ and Barrett esophagus.^[56]^ In this present study, GDF7 was significantly down regulated in GC while a higher GDF7 expression was correlated with significant poorer OS and DFS in patients with GC.

The expression of GDF9 is also heterogeneous in different tumors. It had a reduced or loss of expression in kidney cancer,^[57]^ while it is positive regulated in prostate cancer cells.^[58]^ Our analysis found GDF9 is highly expressed in GC cells. A higher GDF9 expression was correlated with poor OS and DFS in all of the GC patients, but with no significance.

GDF10 is the target of variety tumor therapies including triple-negative breast cancer,^[59]^ prostate cancer,^[60]^ hepatocellular carcinoma,^[61]^ lung cancer,^[62]^ and oral squamous cell carcinoma.^[63]^ GDF10 can be regulated by lncRNA ZFPM2-AS1 to enhance tumor invasiveness in hepatocellular carcinoma.^[61]^ GDF10 was significantly up-regulated in GC tissue. And the higher the expression of GDF10 would bring the shorter DFS rate for GC patients.

GDF11 is a member of the super family of TGF-β and a subfamily of the BMP which is widely secreted in many species.^[64]^ The expression of DGF11 in different tumor tissues is heterogeneous. GDF11 has inhibitory effects on many kinds of tumors, such as liver cancer and breast cancer.^[65,66]^ And GDF11 is also downregulated in pancreatic cancer and other tumors.^[25]^ On the other hand, DGF11 is highly expressed in colorectal cancer and uveal melanoma.^[67,68]^ The methylation level of GDF11 in GC decreased significantly, which made the expression of GDF11 signally up-regulated. However, there was no significant difference in survival curve between GC patients between higher expression of GDF11 and lower counterparts.

In nutshell, we systematically analyzed the expression and prognostic value of GDFs in GC, and we provided a thorough understanding of the heterogeneity and complexity of the molecular biological properties for GC in this research. This exploration confirmed that the GFDs family played a crucial role in GC oncogenesis Our results indicated that GDF1, 3, 6, 7, 10, 15 could be considered as potential tumor biomarker for the diagnosis of GC patients. Our findings suggested that high GDF1, 5, 7, and 15 expressions could also serve as molecular markers to identify high-risk subgroups of GC patients. And GDF3 were potential therapeutic targets for GC, as well as GDF6. Last but not least, GDF1, 3, 6, 7, and 15 could be considered as potential tumor biomarker for GC patients’ prognosis evaluation.

## Author contributions

**Conceptualization:** Minjie Zhu, Jiawei Hong, Xianfang Liu.

**Data curation:** Minjie Zhu, Jiawei Hong.

**Formal analysis:** Minjie Zhu, Jiawei Hong, Haiming Wang.

**Investigation:** Haiming Wang.

**Project administration:** Longquan Lou.

**Writing – original draft:** Minjie Zhu, Jiawei Hong, Longquan Lou.

**Writing – review & editing:** Jiawei Hong.
